# PRMT5 Maintains Homeostasis of the Intestinal Epithelium by Modulating Cell Proliferation and Survival

**DOI:** 10.1002/advs.202415559

**Published:** 2025-02-03

**Authors:** Leilei Li, Zhe Zhang, Xu Wang, Haiyong Zhao, Liansheng Liu, Yanhui Xiao, Shan Hua, Ye‐Guang Chen

**Affiliations:** ^1^ Guangzhou Laboratory Guangzhou 510700 China; ^2^ The State Key Laboratory of Membrane Biology Tsinghua‐Peking Center for Life Sciences School of Life Sciences Tsinghua University Beijing 100084 China; ^3^ School of Basic Medicine Jiangxi Medical College Nanchang University Nanchang 330031 China

**Keywords:** cell proliferation and survival, intestinal homeostasis, protein arginine methylation, protein arginine methyltransferases 5, stem cells

## Abstract

Intestinal homeostasis is sustained by self‐renewal of intestinal stem cells, which continuously divide and produce proliferative transit‐amplifying (TA) and progenitor cells. Protein arginine methyltransferases 5 (PRMT5) plays a crucial role in regulating homeostasis of various mammalian tissues. However, its function in intestinal homeostasis remains elusive. In this study, conditional knockout of Prmt5 in the mouse intestinal epithelium leads to a reduction in stem cell population, suppression of cell proliferation, and increased cell apoptosis within the intestinal crypts, accompanied with shortened gut length, decreased mouse body weight, and eventual animal mortality. Additionally, Prmt5 deletion or its enzymatic inhibition in intestinal organoids in vitro also shows resembling cellular phenotypes. Methylome profiling identifies 90 potential Prmt5 substrates, which are involved in RNA‐related biological processes and cell division. Consistently, Prmt5 depletion in intestinal organoids leads to aberrant alternative splicing in a subset of genes related to the mitotic cell cycle. Furthermore, Prmt5 loss triggers p53‐mediated apoptosis in the intestinal epithelium. Collectively, the findings uncover an indispensable role of PRMT5 in promoting cell proliferation and survival, as well as maintaining stem cells in the gut epithelium.

## Graphical abstract

Cell proliferation and survival are indispensable for intestinal stem cell renewing and the maintenance of intestinal epithelial homeostasis. It is revealed that Protein arginine methyltransferases 5 (PRMT5) is essential for intestinal epithelial homeostasis by sustaining cell proliferation and survival. The finding sheds light on protein arginine methylation in the regulation of gut homeostasis.

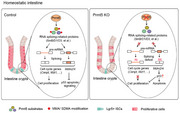

## 1 Introduction

The small intestinal epithelium is organized into crypt‐villus units, whereas the large intestine lacks villus structures. Remarkably, the intestinal epithelium undergoes rapid renewal, with a turnover time of 3–5 days, making it the fastest self‐renewing tissue in mammals.^[^ ^1^ ^]^ The constant renewal of the intestine epithelium is fueled by LGR5^+^ intestinal stem cells (ISCs) located at the base of the crypts.^[^ ^2^ ^]^ These ISCs undergo continuous division, giving rise to rapidly dividing transit‐amplifying (TA) cells and proliferative progenitors.^[^ ^3^ ^]^ These daughter cells then migrate out of the crypt compartment and upward the villus as they mature into differentiated secretory cells (Paneth cells, goblet cells, enteroendocrine cells, and tuft cells) or absorptive enterocytes.^[^ ^2^, ^4^ ^]^ Lgr5^+^ stem cells divide approximately every 24 h, while TA cells complete their four to five cell cycles in even less time. Cell proliferation and its regulations are indispensable for the maintenance of intestinal homeostasis.^[^ ^4^, ^5^ ^]^

Protein arginine (R) methylation is a prevalent post‐translational modification and functions in varieties of biological processes.^[^ ^6^ ^]^ In mammals, this modification is catalyzed by nine members of the protein arginine methyltransferase (PRMT) family.^[^ ^7^ ^]^ PRMTs can be classified into three categories according to their catalytic activity. Type I enzymes (PRMT1, PRMT2, PRMT3, CARM1, PRMT6, and PRMT8) catalyze the formation of ω‐N^G^‐monomethylarginine (MMA) and ω‐N^G^, N^G^‐asymmetric dimethylarginine (ADMA), type II ones (PRMT5 and PRMT9) catalyze the formation of MMA and ω‐N^G^, N^G^‐symmetric dimethylarginine (SDMA), while the type III enzyme PRMT7 only carries out the formation of MMA.^[^ ^8^ ^]^

Numerous reports demonstrate the crucial roles of PRMTs in the regulation of embryonic development or adult tissue homeostasis.^[^ ^8^, ^9^ ^]^ For instance, PRMT5 has been shown to regulate neural stem cell self‐renewal and oligodendrocyte differentiation in the nervous system.^[^ ^10^ ^]^ It is also essential for the long‐term maintenance of muscle stem cells and injury‐induced regeneration,^[^ ^11^ ^]^ as well as contractile function of skeletal muscles.^[^ ^12^ ^]^ In the reproductive system, PRMT5 is involved in spermatogonial stem cell maintenance and required for ovarian follicle development.^[^ ^13^ ^]^ Furthermore, PRMT5 is important for maintaining the quiescence and viability of hematopoietic stem cells and sustains normal adult hematopoiesis.^[^ ^14^ ^]^ Finally, it has also been shown to regulate lipid droplet biogenesis in white adipose tissues and insulin expression in pancreatic islets.^[^ ^15^ ^]^ However, the role of PRMT5 in the maintenance of intestinal homeostasis remains unrevealed.

In the present study, we show that PRMT5 is required for the maintenance of the intestinal epithelium. Deletion of PRMT5 decreases stem cells and suppresses cell proliferation in the epithelium. PRMT5‐methylome profiling reveals a number of potential substrates that associate with the mRNA processing or cell division, which is supported by the observation that PRMT5 loss leads to disrupted alternative pre‐mRNA splicing of a subset of cell cycle‐related genes. Furthermore, we show that Prmt5 deficiency triggers activation of the p53 apoptotic pathway.

## 2 Results

### 2.1 Prmt5 is Required for Intestinal Homeostatic Maintenance

**Figure 1:**
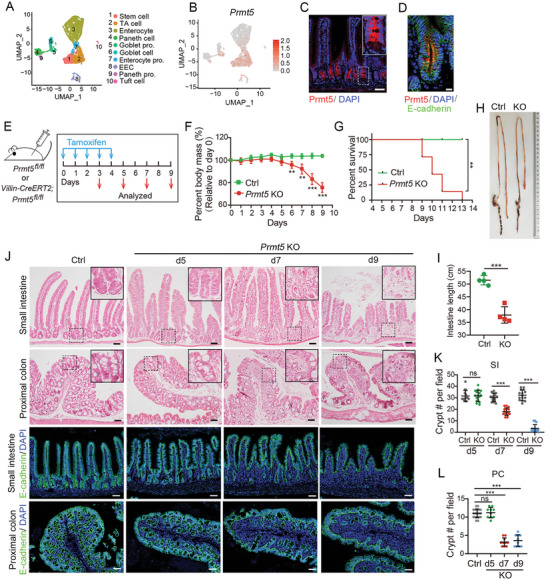
Prmt5 is required for intestinal homeostatic maintenance. A) Cell‐type clusters of small intestinal epithelium were visualized by UMAP in scRNA‐seq analysis of small intestinal epithelium. Paneth pro., Paneth progenitor; EEC, Enteroendocrine cell; Enterocyte pro., Enterocyte progenitor; Goblet pro., Goblet progenitor. B) The expression of Prmt5 in different cell types was visualized by UMAP in scRNA‐sequencing analysis of small intestinal epithelium. C,D) Immunofluorescence staining of Prmt5 and E‐cadherin in small intestinal epithelium. E) Schematic of tamoxifen treatment regimen. Control (*Prmt5^fl/fl^*) and *Prmt5* KO (*Villin‐CreERT2; Prmt5^fl/fl^*) mice were sacrificed and analyzed at indicated time points. F) Body mass following TAM treatment. n = 3–7 mice/group. The body mass at individual day was compared. G) Survival curve of mice following TAM treatment. n = 7 mice/group. H,I) Intestine image and intestinal length of Control and *Prmt5* KO mice. n = 4 mice/group. J) H&E staining and E‐cadherin staining in small intestine and proximal colon at the indicated time points. K,L) Quantification of crypt number in small intestine (K) and proximal colon (L) at indicated time points. K, n = 15–16 random fields from 3 mice/group; L, n ≥ 10 random fields from 3 mice/group. SI, small intestine; PC, proximal colon. All the data represent mean ± SD. ***p < 0.001, **p < 0.01, *p < 0.05, ns = not significant, unpaired student‐t test (F, I), Log‐rank (Mantel‐Cox) test (G), Mann‐Whitney (two‐tailed) U‐test (K, L). Scale bars: 50 µm (C, J), 10 µm (D). Nuclei were counter‐stained with DAPI.

To investigate the role of protein arginine methylation in regulating the intestinal epithelium homeostasis, we characterized the expression pattern of Prmt family members in mouse small intestinal epithelium using a published single‐cell RNA dataset,^[^ ^3b^ ^]^ and found that Prmt1, Prmt3, Carm1, Prmt5, and Prmt7 exhibited high expression levels in the majority of TA and stem cells, with relatively lower expression in progenitor cells (**Figure** **1**A,B; Figure S1A,B, Supporting Information). Conversely, other Prmt members showed minimal expression in the epithelial cells. As Prmt5 protein was present at all segments of small intestine as well as the proximal colon and enriched at the crypt region of the epithelium, especially in Lgr5^+^ ISCs (Figure 1C,D; Figure S1C‐E, Supporting Information), we focused on Prmt5.

To further investigate the role of Prmt5 in intestinal epithelium, we generated inducible *Prmt5* conditional knockout (KO) *Villin‐CreERT2; Prmt5^fl/fl^* mice (Figure 1E). Tamoxifen‐induced deletion of *Prmt5* in intestinal epithelium caused a rapid‐onset body weight loss and shortened intestine length, with KO mice dying in 9–13 days (Figure 1F‐I). Prmt5‐deficiency resulted in a highly disorganized structure and nearly complete loss of the crypts in the small intestine and proximal colon displayed at day 7 or 9 post‐tamoxifen treatment (dpt), while the distal colon exhibited a less severe phenotype (Figure 1J‐L; Figure S1F, Supporting Information). Meanwhile, Prmt5 staining was markedly decreased at 3 dpt and nearly undetectable at 5 dpt (Figure S1G, Supporting Information). Taken together, these data reveal an essential role of Prmt5 in the maintenance of the intestinal epithelium.

### 2.2 Prmt5 Deficiency Results in Impaired Cell Proliferation and Loss of Stem Cells in the Intestinal Epithelium

**Figure 2:**
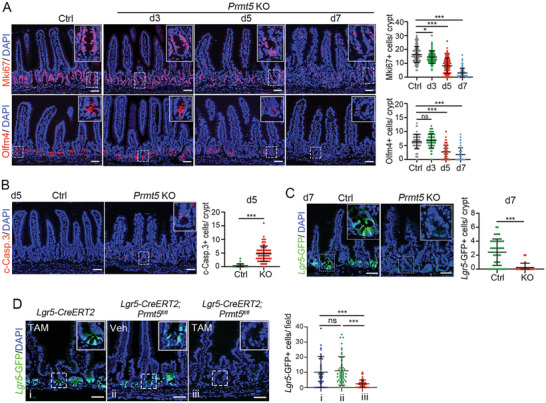
Prmt5 is essential for cell proliferation and stem cell maintenance in intestinal epithelium. A) Immunofluorescence staining images and quantification of Mki67^+^ or Olfm4^+^ cells in small intestinal epithelium at indicated time points. Upper, 69–121 random crypts from 2–3 mice/group were counted; Lower, n = 152–169 random crypts from 3 mice/group were counted. B) Immunofluorescence staining images and quantification of c‐Casp.3^+^ cells in small intestinal epithelium at 5 dpt. n = 42–73 random crypts from 3 mice/group. C) Immunofluorescence images and quantification of *Lgr5*‐GFP^+^ cells in small intestinal epithelium from Control (*Lgr5‐EGFP‐IRES‐CreERT2*; *Prmt5^fl/fl^* treated with corn oil) and Prmt5 KO (*Villin‐CreERT2*; *Lgr5‐EGFP‐IRES‐CreERT2*; *Prmt5^fl/fl^* treated with TAM) mice at 7 dpt. n = 31–45 random crypts from 2–3 mice/group. D) Immunofluorescence images and quantification of *Lgr5*‐GFP^+^ cells in small intestinal epithelium from *Lgr5‐EGFP‐IRES‐CreERT2* (*Lgr5‐CreERT2*) control mice treated with TAM or *Lgr5‐EGFP‐IRES‐CreERT2*; *Prmt5^fl/fl^* (*Lgr5‐CreERT2*; *Prmt5^fl/fl^*) control mice treated with corn oil, and Prmt5 ISC‐specific KO mice (*Lgr5‐CreERT2*; *Prmt5^fl/fl^*) treated with TAM. n = 48 random fields from 2–3 mice/group. All the data represent mean ± SD. ***p < 0.001, **p < 0.01, *p < 0.05, ns = not significant, Mann‐Whitney (two‐tailed) U‐test. Scale bars: 50 µm. Nuclei were counter‐stained with DAPI.

Then we assessed the effect of Prmt5 deletion on cell composition of the intestinal epithelium. Immunofluorescence staining showed that in Prmt5‐KO small intestine, the number of proliferative cells, marked by Mki67, began to decrease as early as 3 dpt, and progressively reduced afterwards (**Figure** **2**A). Similarly, the number of stem cells, marked by Olfm4, also showed a gradual reduction at 5 or 7 dpt (Figure 2A). In the proximal colon, we also observed a dramatic decrease of Mki67^+^ cells at 5 or 7 dpt (Figure S2A, Supporting Information). Simultaneously, apoptotic cells marked by cleaved caspase‐3 displayed a prominent increase in the crypts of either small intestine or proximal colon, as detected at 5 dpt, respectively (Figure 2B; Figure S2B, Supporting Information). However, there were no significant changes in the number of lysozyme^+^ Paneth cells, Mucin2^+^ goblet cells, Chga^+^ enteroendocrine cells, Dclk1^+^ tuft cells, as well as Fabp1^+^ enterocytes (Figure S2C, Supporting Information), indicating that Prmt5 is essential for cell proliferation and stem cell maintenance, rather than for the post‐mitotic differentiated cells.

To further evaluate the effect of Prmt5 on the stem cells, we generated *Villin‐CreERT2; Lgr5‐EGFP‐IRES‐CreERT2; Prmt5 ^fl/fl^* mice, in which ISCs were labeled by *Lgr5*‐GFP. Consistently, the number of *Lgr5*‐GFP^+^ ISCs was remarkably diminished in small intestinal or proximal colonic epithelium upon Prmt5 depletion (Figure 2C; Figure S2D, Supporting Information). Furthermore, the specific knockout of Prmt5 in ISCs using *Lgr5‐EGFP‐IRES‐CreERT2; Prmt5 ^fl/fl^* mice also showed a similar result (Figure 2D). Taken together, the data indicate that Prmt5 plays an indispensable role in promoting cell proliferation and survival and sustaining intestinal stem cells.

### 2.3 Prmt5 is Necessary for Cell Proliferation and Survival in Intestinal Organoids

**Figure 3:**
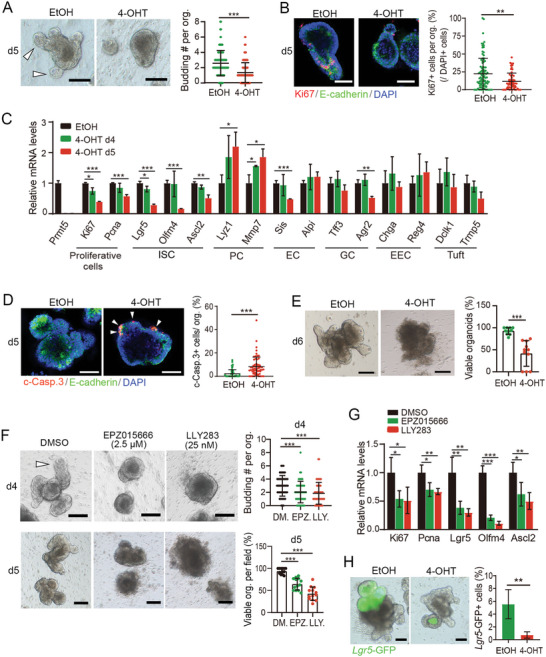
Prmt5 is necessary for cell proliferation and survival in intestinal organoids. A) Images and quantification of budding in small intestinal organoids (*Villin‐CreERT2*; *Prmt5^fl/fl^*) 5 days following EtOH (vehicle) or 4‐ OHT treatment. Arrowheads show bud. n = 104–107 organoids from 10 random fields (2‐3 replicates) per group. Data were from one of three independent experiments. B) Immunofluorescence staining of Mki67 and E‐cadherin in the organoids 5 days following indicated treatments. Percentage of Mki67^+^ cells (relative to DAPI^+^ cells) was quantified. n = 60–101 organoids from 9 random fields/group. Data were from one of two independent experiments. C) The relative expression levels of indicated marker genes were assessed by RT‐qPCR in the organoids at day 4 and 5 following EtOH or 4‐OHT treatment. Data were from one of three independent experiments. D) Immunofluorescence staining of c‐Casp.3 and E‐cadherin in the organoids 5 days following EtOH or 4‐OHT treatment. Percentage of c‐Casp.3^+^ cells (relative to DAPI^+^ cells) was quantified. n = 63–64 organoids from 6–9 random fields/group. Data were from one of two independent experiments. E) Images and quantification of viable organoids (relative to total organoids/ field) 6 days following EtOH or 4‐OHT treatment. n > 10 fields/group. Data were from one of three independent experiments. F) Images and quantification of budding number or viable organoids (relative to total organoids/ field), following 4‐day or 5‐day treatment with compounds, respectively. Viable organoids were identified by the presence of a distinct and intact epithelial cell border. Organoids with a dark appearance, broken and indistinguishable cell borders throughout the entire body were considered non‐viable. Arrowhead shows bud. n = 50–88 organoids from 8–10 random fields (2–3 replicates) per group (d4); n > 10 fields/group (d5). Data were from one of three independent experiments. G) The relative expression levels of indicated marker genes were detected by RT‐qPCR in organoids following compound treatment. Data from four independent experiments were combined and are shown. H) Small intestinal organoids (*Lgr5‐EGFP‐IRES‐CreERT2*; *Prmt5^fl/fl^*) were treated with EtOH or 1 μΜ 4‐OHT for 2 days and the *Lgr5*‐GFP^+^ cells were visualized and quantified by flow cytometry at day 10 post treatment. All the data represent mean ± SD. ***p < 0.001, **p < 0.01, *p < 0.05, Mann‐Whitney (two‐tailed) U‐test (A, B, D‐F), unpaired student‐t test (C, G, H). Scale bars: 50 µm (B, D), 100 µm (A, E, F), 200 µm (H). Nuclei were counter‐stained with DAPI.

Next, we further assessed the role of Prmt5 in intestinal epithelium in in vitro cultured organoids. The small intestinal organoids were established from crypts derived from *Villin‐CreERT2; Prmt5^fl/fl^* mice, and deletion of Prmt5 was achieved in the organoids by the treatment of 4‐hydroxytamoxifen (4‐OHT) (Figure S3A,B, Supporting Information). Upon Prmt5 knockout, the number of organoid budding and Mki67^+^ cells were significantly decreased (**Figure** **3**A,B); the mRNA levels of proliferative or stem cell marker genes were gradually downregulated, while the expression of most differentiated cell markers remained unchanged (Figure 3C). Notably, Prmt5‐deficient organoids displayed dramatically increased c‐Casp.3^+^ apoptotic cells (Figure 3D), and eventually underwent death (Figure 3E; Figure S3C, Supporting Information). In addition, treating organoids with Prmt5 enzymatic inhibitor EPZ015666 or LLY283 also suppressed organoid budding (Figure 3F), reduced the expression of proliferative and stem cell markers (Figure 3G), and induced cell death (Figure 3F; Figure S3D, Supporting Information). Furthermore, the specific knockout of Prmt5 in *Lgr5*‐GFP^+^ ISCs in organoids led to a remarkable decrease of the Lgr5^+^ cells (Figure 3H; Figure S3E, Supporting Information), reinforcing the critical role of Prmt5 in ISCs. Together, these results further demonstrate the essential role of Prmt5 in promoting cell proliferation and survival, therefore maintaining intestinal stem cells.

### 2.4 Methylome Profiling Reveals Potential Prmt5 substrates in the intestinal epithelium

**Figure 4:**
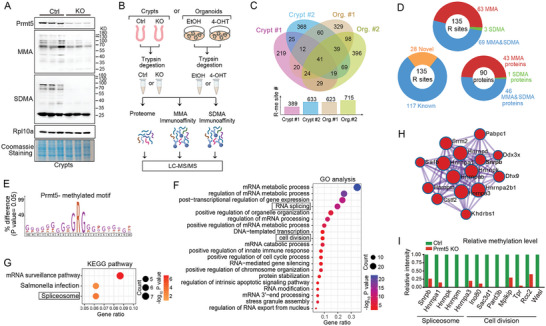
Methylome profiling reveals potential Prmt5 substrates. A) The global MMA or SDMA was immunoblotted in small intestinal crypts isolated from Control (*Prmt5^fl/fl^*) and *Prmt5* KO (*Villin‐CreERT2*; *Prmt5^fl/fl^*) mice at 3 dpt. Rpl10a immunobolt and coomassie‐stained protein bands represent normalized total protein level. B) Outline of the proteome and methylome profiling strategy. Two independent experiments were conducted in both isolated crypts and in vitro cultured organoids, for a total of four experiments. For each independent assay in crypts, the small intestinal crypts were isolated from three Prmt5*^fl/fl^* mice (control) and three *Villin*‐creERT2; Prmt5*^fl/fl^* mice (KO) following TAM treatment. The crypts from the three mice in each group were pooled together for methylome profiling in the control or KO group. For each independent assay in organoids, small intestinal organoids (*Villin*‐creERT2; Prmt5*^fl/fl^*) treated with either EtOH or 4‐OHT were collected and subjected to methylome profiling. For individual profiling assays, the control or KO, EtOH or 4‐OHT group underwent MMA, SDMA, and proteome analysis by label‐free LC‐MS/MS. C) Venn diagram shows the number of identified differential methylated‐R (R‐me) sites with at least twofold decrease in their peptide abundances upon *Prmt5* KO in the four independent profiling assays. Upper, overlap among four independent assays; lower, differential R‐me number in four assays. The diagrams were plotted by an online tool E‐Venn. D) Pie charts show the overlapped differential methylated‐R sites and corresponding proteins. Upper, the number of R sites with different modification forms; lower, the number of known or novel methylated R sites (left) and the number of proteins with different modification types (right). Known arginine methylated sites (mouse) were retrieved from www.phosphosite.org ^[^ ^34^ ^]^ E) Motif analysis was performed for PRMT5‐methylated arginine sites using IceLogo. F‐H) Gene ontology (GO) (F), KEGG pathway (G), and Protein‐Protein interaction (H) of potential Prmt5 substrates were analyzed using Metascape. Protein‐Protein interaction network involved in mRNA metabolic process, mRNA processing, and RNA splicing is shown. I) Relative methylation levels of indicated proteins in Control and *Prmt5* KO group. The methylation level of individual protein represents the average abundance of methylated peptides from four methylome profilings.

We observed that Prmt5 deficiency led to a dramatic reduction of the global protein arginine methylation monomethylarginine (MMA) or symmetric dimethylarginine (SDMA) in isolated intestinal crypts or organoids (**Figure** **4**A; Figure S4A, Supporting Information), indicating a regulation of Prmt5 on the global protein arginine modification in the intestinal epithelium. To identify the potential substrates, we performed methylome profiling assays by label‐free liquid chromatography‐tandem mass spectrometry (LC‐MS/MS)‐based proteomics with the samples of either isolated intestinal crypts from control or Prmt5 KO mice, or in vitro cultured small intestinal organoids (Figure 4B).

From four independent Prmt5‐methylome analyses (two independent assays were conducted for each pair of control/ KO crypt or organoid samples), we identified 623–1015 MMA sites (Figure S4B and Table S1, Supporting Information) and 170–322 SDMA sites (Figure S4C and Table S1, Supporting Information) in the control group, uncovering an arginine modification pattern in the homeostatic intestinal epithelium. To ensure the accuracy and consistency, we selected the methylated R (R‐me) sites present in at least three independent assays. This resulted in 374 R sites (327 with MMA, 16 with SDMA, and 31 with both MMA and SDMA), corresponding to 197 proteins (Figure S4D, Tables S2.1 and S2.2, Supporting Information). Notably, among the identified R‐me sites, 90 were novel (Figure S4D and Table S2.5, Supporting Information). Gene Ontology (GO) and Kyoto Encyclopedia of Genes and Genomes (KEGG) pathway enrichment analyses revealed that these arginine‐methylated proteins function in RNA biology, including mRNA metabolic process, mRNA splicing and surveillance and RNA localization, as well as other biological processes such as post‐transcriptional regulation of gene expression, regulation of organelle organization and cellular response to stress (Figure S4E,F, Supporting Information). Interestingly, we noticed an enrichment on stem cell population maintenance (Figure S4E,G, Supporting Information), suggesting a possible role of protein arginine methylation in the regulation of intestinal stem cells.

**Figure 5:**
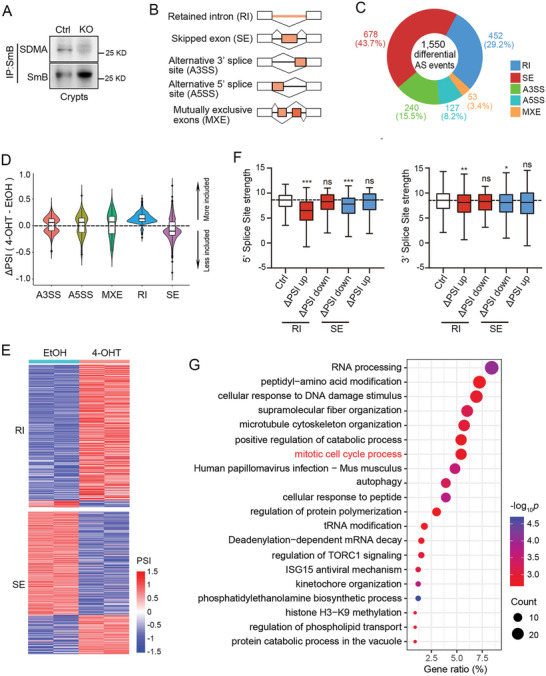
Prmt5 deletion leads to changes in alternative splicing. A) SmB (Snrpb) protein was immunoprecipitated and the SDMA modification was detected by immunoblotting in small intestinal crypts isolated from Control or *Prmt5* KO mice. B) The schematic diagram shows several alternative splicing events. The orange line represents retained intron and the orange box skipping exon. C) The *Villin‐CreERT2*; *Prmt5^fl/fl^* organoids were treated with EtOH or 4‐OHT, with two biological replicates per group, and subjected to bulk RNA‐sequencing and alternative splicing analysis. Pie chart shows differential alternative splicing (AS) events between two groups. The number of different event types and their percentage are shown. D) Violin plot shows ΔPSI values of the differential AS events in organoids. Δ PSI = PSI (4‐OHT)‐ PSI (EtOH). Values above the dashed line represent increased intron or exon inclusion upon 4‐OHT treatment, while values below the line represent decreased intron or exon inclusion. E) Heatmap shows the PSI values (scaled by Z‐score) in RI and SE events in *Villin‐CreERT2*; *Prmt5^fl/fl^* organoids treated with EtOH or 4‐OHT. Two biological replicates were used per group. F) 5′ and 3′ splice site strength in RI or SE events were scored by MaxEntScan using the maximum entropy scoring model. The splice site scores in PSI‐unchanged events were used as the control group. Tukey boxplots representing splice site scores are shown. ***p < 0.001, **p < 0.01, *p < 0.05, ns = not significant, unpaired student t‐test. G) GO&KEGG enrichment analyses of genes in the differential RI and SE events. Top 20 terms are shown.

Then, we surveyed the differentially methylated R sites upon Prmt5 loss. To exclude that the arginine modification changes were just due to the alterations of the protein expression, relative abundances of modified peptides were normalized to their corresponding protein levels. From the four independent analyses, we obtained 389–715 methylated R sites with at least twofold decrease in their peptide abundances upon Prmt5 KO (Figure 4C; Table S1, Supporting Information). We then selected the overlapped differentially modified R sites present in at least three profiling assays, and this generated 135 R sites (63 with MMA, 3 with SDMA, and 69 with both MMA and SDMA, 28 novel), related to 90 proteins that were defined as the potential Prmt5 substrates (Figure 4D; Tables S2.3, S2.4, and S2.5, Supporting Information). Motif analysis revealed a consensus GAR (glycine and arginine) motif for Prmt5‐methylated arginine sites (Figure 4E). Notably, the reported PRMT5 substrates, such as Snrpb,^[^ ^16^ ^]^ Fubp1,^[^ ^17^ ^]^ Ripk3,^[^ ^18^ ^]^ Rps10,^[^ ^19^ ^]^ Hnrnpa1^48^ and Znf326,^[^ ^20^ ^]^ were identified. Moreover, we selected several potential substrates to validate their methylation by PRMT5 in the cells. Except for Hnrnpdl that had no signals, others showed dramatically decreased MMA or SDMA levels upon Prmt5 KO or inhibition (**Figure** **5**A; Figures S4H,I and S5B, Supporting Information), supporting the accuracy of our profiling. Further, GO and KEGG analyses of the set of 90 potential substrates also revealed a prominent involvement in RNA biology‐related processes, like RNA splicing and spliceosome. Of note, cell division was also enriched (Figure 4F‐H). Supporting it, the methylation levels of the substrates related to cell division and spliceosome were greatly reduced in the Prmt5 KO intestinal epithelium (Figure 4I).

Notably, the expression of other Prmts did not show obvious changes either in their mRNA levels (Figure S4J,K, Supporting Information) or in their protein abundance (Figure S4L, Supporting Information) upon Prmt5 deletion in intestinal epithelium or organoids. Furthermore, Prmt2, Prmt6, Prmt8 or Prmt9 were nearly undetectable in their mRNA or protein levels in our context. Therefore, the phenotypes we observed were due to PRMT5 deficiency in the Prmt5 KO mice.

### 2.5 Prmt5 Deletion Leads to Changes in Alternative Splicing

It is wellknown that PRMT5 symmetrically dimethylates several Sm proteins (i.e., SmB, SmD1 or SmD3) to facilitate the biogenesis of spliceosomal snRNPs, essential factors for pre‐mRNA splicing.^[^ ^21^ ^]^ In agreement, we observed the markedly reduced methylation of SmB (Snrpb) upon Prmt5 loss (Figure 4I). In addition, we validated Prmt5‐mediated SDMA modification of SmB, SmD1 or SmD3 proteins in intestinal crypts or colonic HCT116 cells (Figure 5A; Figure S5A‐C, Supporting Information). To further investigate the role of Prmt5 in modulating pre‐mRNA splicing, we analyzed alternative pre‐mRNA splicing with our bulk RNA sequencing data by rMATS analysis.^[^ ^22^ ^]^ We detected 1550 differential alternative splicing (AS) events (distributed in 1197 genes), encompassing retained intron (RI), skipped exon (SE), alternative 3′ splice sites (A3SS), alternative 5′ splice sites (A5SS), and mutually exclusive exons (MXE), with the majority being RI and SE events (Figure 5B,C; Table S3, Supporting Information). In Prmt5 KO group, the vast majority of RIs exhibited upregulated percent‐spliced‐in (PSI, ratio of the long isoform relative to the sum of long and short isoforms in the transcripts of a gene) values (Figure 5D,E), suggesting the tendency of Prmt5‐deficient organoids to retain introns in mRNA molecules. Conversely, over half of the SE events showed downregulated PSI values (Figure 5D,E), indicating a propensity for exon loss in mRNA transcripts. Thus, these results suggest that the splicing efficiency is compromised following Prmt5 loss. Indeed, the splice junctions affected by Prmt5 depletion commonly featured weaker 5′ or 3′ splice sites, particularly in the case of upregulated RI events, which exhibited substantially diminished 5′ splice site strength (Figure 5F; Table S4, Supporting Information). Furthermore, GO and KEGG enrichment analyses of RI‐ and SE‐ associated genes (393 and 580 genes, respectively) revealed the major involvement in processes including RNA processing, peptidyl‐amino acid modification, response to DNA damage stimulus, and notably, mitotic cell cycle (Figure 5G). Nevertheless, we observed a limited overlap between the differentially spliced and differentially expressed genes (Figure S5D, Supporting Information), indicating that the identified alternative splicing changes have little impact on total mRNA expression levels.

### 2.6 Prmt5 Loss Disrupts Splicing of a Subset of Mitotic Cell Cycle‐Related Genes

**Figure 6:**
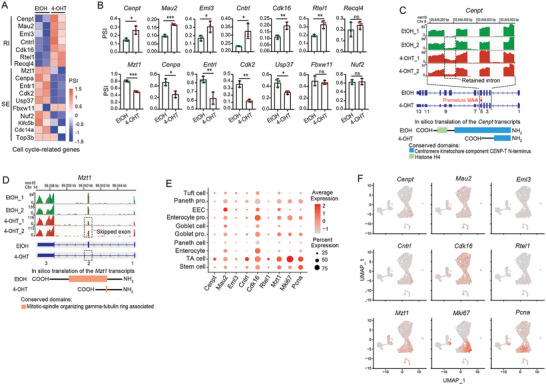
Prmt5 loss disrupts splicing of a subset of mitotic cell cycle‐related genes. A) Heatmap shows the PSI values (scaled by Z‐score) of cell cycle‐related genes in RI and SE events in organoids. Two biological replicates for EtOH or 4‐OHT group. B) Disrupted RI and SE events in cell cycle genes were validated by RT‐PCR in *Villin‐CreERT2*; *Prmt5^fl/fl^* organoids treated with EtOH or 4‐OHT. Three biological replicates/group. The intensity of every band in Figure S6A (Supporting Information) was quantified by ImageJ. And PSI was calculated based on band intensity of long isoform and short isoform in every replicate per group. Data was from one of three independent experiments. ***p < 0.001, **p < 0.01, *p < 0.05, ns = not significant, unpaired student t‐test. C,D) Schematic representation of the differential alternative splicing events in *Cenpt* (C) and *Mzt1* (D) RNA transcripts. The reads from RNA‐sequencing and the corresponding chromosomal positions are visualized in Integrative genomics viewer (IGV). Dashed box outlines the differential RI or SE event. Protein conserved domains were found in CDD database in NCBI. E) Dot plot of cell cycle gene expression in different cell types in scRNA‐sequencing analysis of small intestinal epithelium. F) Expression of cell cycle‐related gene were visualized by UMAP in scRNA‐sequencing analysis. Paneth pro., Paneth progenitor; EEC, Enteroendocrine cell; Enterocyte pro., Enterocyte progenitor; Goblet pro., Goblet progenitor.

Next, we further investigated Prmt5 loss‐induced splicing changes of the mitotic cell cycle‐related genes in intestinal organoids. There were 7 genes in RI events and 10 genes in SE events, exhibiting increased and decreased inclusion, respectively, upon Prmt5 KO in the rMATS analysis (**Figure** **6**A). To validate the result, we conducted RT‐PCR and found that alternative splicing in 14 out of 17 (82%) events were confirmed (Figure 6B; Figure S6A, Supporting Information). Notably, 11 of these validated events (6 RIs and 5 SEs) demonstrated significantly increased intron retention or exon skipping in response to Prmt5 depletion (Figure 6B). Subsequently, we illustrated the potential functional implication of these Prmt5‐affected splicing events. In the case of the gene *Cenpt*, differential intron inclusion between exon 4 and 5 resulted in the generation of a premature termination codon (PTC) within exon 5 of the transcript (Figure 6C). The PTCs were also observed in five other affected retained introns (*Mau2*, *Eml3*, *Cntrl*, *Cdk16*, and *Rtel1*), potentially instigating nonsense‐mediated mRNA decay (NMD) and/or truncation of the protein products, as previously reported.^[^ ^23^ ^]^ In the *Mzt1* gene, exon 2 skipping led to the loss of a substantial portion of its coding region, resulting in a significant truncation of the functional protein domain (Figure 6D). However, Prmt5‐regulated SEs in other genes like *Cenpa*, *Entrl*, *Cdk2* and *Usp37* primarily resulted in changes in the composition of known transcript variants, with the functional consequence remaining unknown. Given the pivotal roles of these genes in the cell cycle (Table S5, Supporting Information),^[^ ^24^ ^]^ it is conceivable that splicing dysregulation in these genes may compromise normal cell cycle progression. Next, we assessed the expression levels of these functionally affected genes using single‐cell RNA sequencing data from small intestinal epithelium. Nearly all of genes exhibited a relative higher expression in the proliferative cell types (Stem cells, TA and progenitor cells) (Figure 6E,F; Figure S6B, Supporting Information), implying a potential involvement in sustaining cell proliferation in the intestinal epithelium.

### 2.7 Prmt5 Deficiency Activates the p53‐Dependant Apoptotic Pathway

**Figure 7:**
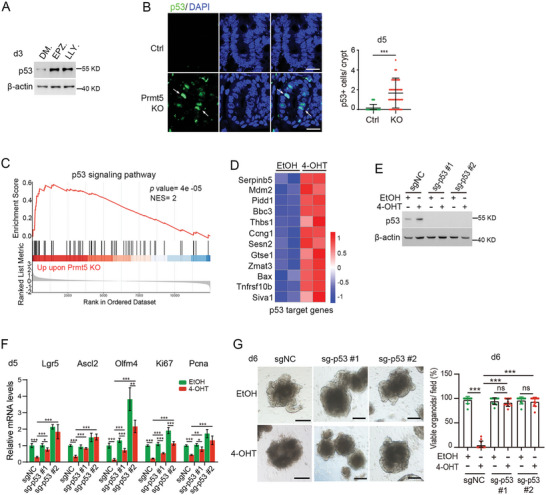
Prmt5 deficiency activates p53‐dependant apoptotic pathway. A) Indicated proteins were detected by immunoblotting in small intestinal organoids treated with vehicle (DMSO), EPZ015666 or LLY283 for 3 days. Data were from one of three independent experiments. B) Immunofluorescence staining images and quantification of p53^+^ cells in small intestinal epithelium at 5 dpt. n = 45 random crypts from 2 mice/group. Nuclei were counter‐stained with DAPI. C) An enrichment of p53 signaling pathway was shown by gene set enrichment analysis (GSEA). D) The heatmap displays p53 target genes that were upregulated (≥ 1.5‐fold) identified by bulk RNA‐sequencing 4 days following EtOH or 4‐OHT treatment in *Villin‐CreERT2*; *Prmt5 ^fl/fl^* organoids. E) The organoids (*Villin‐CreERT2*; *Prmt5 ^fl/fl^*) were transduced with lentivirus carrying Cas9 and negative control sgRNAs (sgNC) or p53‐targeting sgRNAs (sg‐p53 #1 or #2).Then indicated proteins were detected by mmunoblotting in the organoids 4 days following EtOH or 4‐OHT treatment. Data represent one of three independent experiments. F) The stem cell and proliferation marker genes were detected by RT‐qPCR in control or p53 KO organoids (*Villin‐CreERT2*; *Prmt5 ^fl/fl^*) 5 days following EtOH or 4‐OHT treatment. G) Images and quantification of viable organoids (relative to total organoids/ field) in control or p53 KO organoids (*Villin‐CreERT2*; *Prmt5 ^fl/fl^*) 6 days post EtOH or 4‐OHT treatment. n ≥ 10 fields/group. Data were from three independent experiments. All the data represent mean ± SD. ***p < 0.001, **p < 0.01, *p < 0.05, Mann‐Whitney (two‐tailed) U‐test (B, G), unpaired student‐t test (F). Scale bars: 20 µm (B), 200 µm (G).

To further clarify the functional consequence of Prmt5 deficiency, we analyzed the differentially expressed genes in our bulk RNA‐sequencing data in intestinal organoids upon Prmt5 knockout, and we found that downregulated genes mainly enriched in a variety of metabolic processes (Figure S7A and Table S6, Supporting Information). Interestingly, the upregulated genes had an enrichment in cell death, p53 signaling and apoptosis (Figure S7A and Table S6, Supporting Information), consistent with the observed increase in c‐Casp.3^+^ apoptotic cells in intestinal tissues. Furthermore, we observed that *Mdm2* and *Mdm4*, key negative regulators of p53, exhibited increased exon skipping upon Prmt5 KO (Figure S7B,C, Supporting Information). The skipping of exon 3 in *Mdm2* has been reported to result in the absence of its N‐terminus protein product, leading to the loss of its ability to bind p53.^[^ ^25^ ^]^ Exon 7 skipping in *Mdm4* is known to generate a premature termination codon in the transcript and leads to subsequent NMD.^[^ ^21a^ ^]^ Both of aberrant SE events can contribute to p53 upregulation.^[^ ^25^ ^]^ Indeed, we observed elevated p53 protein levels in isolated intestinal crypts (Figure S7D, Supporting Information) or in cultured organoids (**Figure** **7**A,E), as well as marked increase in p53^+^ cells in the intestinal epithelium (Figure 7B), following Prmt5 KO or inhibition. Additionally, the activation of p53 signaling and upregulation of p53 target genes were evident upon Prmt5 loss in organoids (Figure 7C,D). Notably, CRISPR/Cas9‐mediated p53 knockout in organoids substantially rescued the stem cell and proliferation defects caused by Prmt5 loss (Figure 7E,F). Furthermore, p53 knockout reversed Prmt5 loss‐induced organoid death (Figure 7G; Figure S7E, Supporting Information), although the organoids still exhibited mortality during extended passaging (Figure S7F, Supporting Information). Collectively, these results suggest that p53 activation plays a major role in the organoid death triggered by Prmt5 deficiency.

Prmt5 loss is known to induce DNA damage in several mammalian tissues, including fetal hematopoietic stem/progenitor cells and primordial germ cells.^47^ ^[^ ^26^ ^]^ Given that the differentially spliced genes had an enrichment in DNA damage response (Figure 5G), we wondered whether DNA damage occurred in our context and contributed to p53‐mediated apoptosis. However, we only observed a moderate increase in γH2AX, a well‐known marker of DNA damage,^[^ ^27^ ^]^ following Prmt5 inhibition in intestinal organoids (Figure S7G, Supporting Information). As γH2AX can result from DNA fragmentation during cell apoptosis,^[^ ^28^ ^]^ we knocked out p53 to suppress apoptosis, thereby excluding apoptosis‐induced γH2AX signals. Under these conditions, Prmt5 inhibition no longer increased γH2AX levels (Figure S7G, Supporting Information). These findings suggest that the enhanced γH2AX signal is due to cell apoptosis rather than Prmt5 dysfunction, indicating that DNA damage is not responsible for Prmt5 loss‐induced p53 signaling in the intestinal epithelium, consistent with a previous report.^[^ ^14a^ ^]^

## 3 Discussion

**Figure 8:**
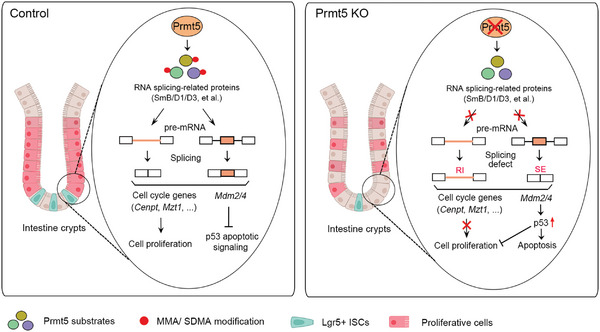
Schematic of Prmt5 in sustaining intestinal cell proliferation and stem cells. In intestinal crypt proliferative cells, including stem cells, Prmt5 methylates various potential substrates including RNA‐splicing related proteins, and is essential for the splicing of a subset of cell cycle‐related gene products or *Mdm2/4* transcripts, which supports cell proliferation or suppresses p53‐mediated apoptosis, respectively. Upon Prmt5 conditional knockout, protein methylation is disrupted, leading to splicing defect in cell cycle genes or *Mdm2/4* transcripts. This results in suppressed cell proliferation and induction of p53‐dependent apoptosis. The intestine models were created with BioRender.com.

The roles of the PRMT family in gut homeostasis are poorly documented so far, except PRMT1 that is reported to be essential for intestinal stem cell formation in gut development and regulates cell proliferation and differentiation in mouse intestine.^46^ ^[^ ^9^ ^]^ Here, we show a high expression of Prmt1/3, Carm1, Prmt5/7 in the proliferative stem cells, TA and progenitor cells in mouse intestinal epithelium, implying a possible role of these PRMT members in regulation of intestinal function. Specifically, we have demonstrated a pivotal role of PRMT5 in maintaining intestinal epithelium homeostasis (**Figure** **8**).

PRMT5 has been reported to play crucial roles in stem cell self‐renewal, cell proliferation, or cell specification and differentiation in several mammalian tissues, including nervous system,^[^ ^10^ ^]^ skeletal muscle,^[^ ^11^ ^]^ hematopoietic cells,^[^ ^14^ ^]^ and ovarian tissue,^[^ ^13b^ ^]^ et al. Here, we show that PRMT5 is essential for cell proliferation and survival and maintenance of intestinal stem cells in the intestinal homeostasis. Importantly, the pronounced effects of Prmt5 deletion exclusively in the proliferative cells, but not in post‐mitotic differentiated cells, underscore the requirement of PRMT5 for cell proliferation in the intestinal epithelium. Given the rapid turnover of the intestinal epithelium,^[^ ^1^, ^29^ ^]^ cell proliferation and survival are extremely important for stem cells’ self‐renewal and continuous supply of progeny TA and progenitor cells for further differentiation of functional epithelium cells.

We identify reliable and reproducible mono‐methylated arginine and symmetrically di‐methylated arginine sites, including a number of novel sites, through four independent methylome profilings in the wild type or PRMT5‐depleted intestinal epithelium, shedding light on the arginine‐methylated non‐histone proteins and the PRMT5‐mediated methylome in intestinal homeostasis. Besides the well‐established RNA biology‐related processes, especially pre‐mRNA splicing, in the identified potential PRMT5 substrates, the cell division‐related ones suggest a possible regulation of PRMT5 in cell proliferation by directly methylating these division‐related regulators. Additionally, substrates involved in other critical cellular processes, such as regulation of gene expression, transcription and chromosome organization, may be involved in PRMT5‐regulated intestinal homeostasis, and the role of their modulation and the underlying molecular mechanisms still need further investigation.

PRMT5 has been shown to modulate alternative splicing of gene products involved in a variety of cellular processes, including γc‐family cytokine signaling,^[^ ^30^ ^]^ DNA damage repair,^[^ ^14^, ^25^ ^]^ cell survival and cell proliferation.^[^ ^31^ ^]^ Here, we identify a novel cohort of mitotic cell cycle‐related genes (*Cenpt*, *Mau2*, *Eml3*, *Cntrl*, *Cdk16*, *Rtel1*, and *Mzt1*) whose splicing fidelity is ensured by PRMT5 in intestinal epithelium. Given the direct involvement of these genes in cell cycle progression, we propose that PRMT5‐mediated correct splicing of these genes contributes to cell proliferation, and, of course, self‐renewal of stem cells in intestinal epithelium. More importantly, these cell cycle genes identified here were not consistently observed in other PRMT5‐depletion or inhibition models. For example, although CDK16 was found to be a target of PRMT5 in acute myeloid leukemia,^[^ ^32^ ^]^ it was not identified in PRMT5‐deleted hematopoietic stem cells.^[^ ^14b^ ^]^ In PRMT5 inhibitor‐treated glioblastoma stem cells, yet a different set of cell cycle genes showed differential splicing.^[^ ^31b^ ^]^ These findings suggest that PRMT5‐affected gene splicing is context‐dependent and varies across different tissue types. Nevertheless, other underlying mechanisms for PRMT5 to regulate cell proliferation and intestinal stem cells couldn't be excluded in our context.

Additionally, we also analyzed the differentially expressed genes from our bulk RNA‐seq data in intestinal organoids upon Prmt5 depletion. Among the enriched cellular functions of the downregulated genes, the metabolic processes related to fatty acids (monocarboxylic acids), vitamin, and amino acids, have reported roles in intestinal stem cell maintenance or differentiation,^[^ ^33^ ^]^ implying that the suppression of these metabolic processes also probably contribute to the observed phenotypes caused by Prmt5 deficiency. However, the molecular mechanism by which PRMT5 regulates these metabolism‐related genes remains unclear.

The p53 induction and cell apoptosis are commonly observed in various mouse tissues when Prmt5 is depleted.^[^ ^14^, ^21^, ^25^, ^26^ ^]^ In line with this, we also observe p53 upregulation and p53 signaling activation in the intestinal epithelium or organoids in the absence of Prmt5. Importantly, the activation of the p53 signaling pathway, maybe triggered by the splicing defects of Mdm2/4 or other potential mechanism, may be a major cause of cell apoptosis induced by Prmt5 deficiency in the intestine. Therefore, the blockage of p53 apoptotic signaling by high expression of Prmt5 in the crypts is essential for the survival and self‐renewal of stem cells in the intestinal epithelium under homeostatic conditions.

In summary, our study uncovers an indispensable role of PRMT5, a PRMT family member, in the maintenance of intestinal epithelial homeostasis via promoting cell proliferation and survival, as well as sustaining intestinal stem cells. Our finding sheds light on protein arginine methylation in the regulation of gut homeostasis.

## 4 Experimental Section

### Mice

*Villin‐CreERT2* mice were kindly provided by Sylvie Robine (Institut Curie, France). *Prmt5 ^fl/fl^* mice were a gift from Fei Gao (Chinese Academy of Sciences, Beijing, China).^[^ ^35^ ^]^ The *Villin‐CreERT2*; *Prmt5^fl/fl^* mice were generated by crossing the above two lines together. *Lgr5‐EGFP‐IRES‐CreERT2* mice were obtained from the Jackson Laboratory, and crossed with *Villin‐CreERT2*; *Prmt5^fl/fl^* animals to generate *Villin‐CreERT2*; *Lgr5‐EGFP‐IRES‐CreERT2*; *Prmt5 ^fl/fl^* or *Lgr5‐EGFP‐IRES‐CreERT2*; *Prmt5 ^fl/fl^* mice. All mouse strains were bred and housed at the animal facility with specific pathogen free (SPF) conditions. All animal studies were performed in accordance with the relevant guidelines, under the approval of the Institutional Animal Care and Use Committee of Guangzhou National Laboratory (approval number: GZLAB‐AUCP‐2023‐01‐A01).

For conditional knockout of *Prmt5* in intestinal epithelium, 8‐10 week‐old *Villin*‐*creERT2; Prmt5^fl/fl^* mice and littermate mice (*Prmt5^fl/fl^*), were intraperitoneally injected with tamoxifen (Sigma, stock 20 mg mL^−1^ in corn oil) at a dose of 100 mg kg^−1^ body mass every 24 h for five consecutive days. For the assessment of *Lgr5*‐GFP^+^ cells in intestinal epithelium, *Villin‐CreERT2*; *Lgr5‐EGFP‐IRES‐CreERT2*; *Prmt5^fl/fl^* mice were treated with tamoxifen using the above regime, and the littermate *Lgr5‐EGFP‐IRES‐CreERT2*; *Prmt5 ^fl/fl^* mice parallelly injected with corn oil were used as the control. For Prmt5‐specific knockout in ISCs, *Lgr5‐EGFP‐IRES‐CreERT2; Prmt5 ^fl/fl^* mice received two rounds of 5‐day tamoxifen or corn oil (control) injections, with a 9‐day interval, and age‐matched *Lgr5‐EGFP‐IRES‐CreERT2* mice parallelly injected with tamoxifen served as an additional control group.

### Intestinal Organoids and Cell Culture

Mouse small intestinal crypts were isolated and cultured in vitro as previously described.^[^ ^36^ ^]^ Briefly, the small intestinal crypts were isolated from 8–10 week‐old *Villin*‐CreERT2; Prmt5*^fl/fl^* mouse, embedded in Matrigel (Corning, #356231) and then seeded on the plate. The crypt‐containing droplets were overlaid with ENR medium (advanced DMEM/F12 supplemented with penicillin/streptomycin, GlutaMax, N2, B27, and N‐acetylcysteine, containing 50 ng mL^−1^ EGF, 100 ng mL^−1^ Noggin, and 500 ng mL^−1^ R‐spondin1) and refreshed every 2–3 days. For ex vivo *Prmt5* depletion, vehicle (ethanol, 1:1000) or 1 µM 4‐OHT (Sigma) was added directly into the medium for 24 h. For Prmt5 enzymatic inhibition, organoids were passaged and recovered for one day, then vehicle (DMSO, 1:1000), 2.5 µM EPZ015666 (MCE, #HY‐12727), or 25 nM LLY283 (MCE, #HY‐107777) was added into the medium for the required time points to analysis.

All quantifications were performed based on the captured images in random fields. Viable organoids were defined by the presence of a distinct and intact epithelial cell border. Organoids exhibiting dark appearance, broken, and undistinguishable cell border in the total body were considered as dead ones. “‘Non‐budding”’ or spheroid organoids were characterized by a round or oval shape without any epithelial protrusions. “Budding” referred to the emergence of an epithelial protrusion representing a crypt from the main spheroid body.

Colon cancer cell line HCT116 or SW480 was cultured in RPMI 1640 or DMEM medium (Gibco), respectively, supplementary with 10% fetal bovine serum (Hyclone) and penicillin/streptomycin.

### Immunofluorescence and H&E Staining

Immunofluorescence and histological staining were conducted as previously described.^[^ ^36^ ^]^ H&E staining was conducted according to the manufacturer instruction (Beyotime, C0205S). The primary antibodies used were as following: rabbit anti‐Prmt5 (1:150, Proteintech, #18436‐1‐AP), mouse anti‐E‐cadherin (1:200, BD Transduction Laboratories, #610182), rabbit anti‐Ki67 (1:200, Abcam, ab15580), rabbit anti‐Olfm4 (1:500, CST, Cat#19141), rabbit anti‐Cleaved Caspase 3 (1:100, CST, #9664L), rabbit anti‐ Lysozyme (1:500, Abcam, ab108508), rabbit anti‐Chga (1:500, Abcam, ab15160), rabbit anti‐Mucin2 (1:1000, Abcam, ab272692), mouse anti‐GFP (1:100, Santa cruz, sc‐9996); Secondary antibodies were goat anti‐mouse IgG, Alexa Fluor 488 (1:1000, ThermoFisher, A11029) and goat anti‐rabbit IgG, Alexa Fluor 594 (1:1000, ThermoFisher, A11037) and DAPI (1:1000, Roche). For p53 staining, mouse anti‐p53 antibody (CST, #2524) was used and the signals were amplified by TYR‐520 dye (Recordbio Biological Technology, Shanghai, China, Cat#RC0086‐1) based on the tyramide signal amplification (TSA) technology according to the manufacture's instruction. Intestinal tissue sections were visualized with a confocal microscope (Zeiss, LSM980).

For organoid staining, the procedure was conducted according to the previous describes.^[^ ^37^ ^]^ Briefly, organoids were collected and fixed with 4% PFA. Primary antibodies were incubated overnight, organoids were washed and secondary antibody was incubated for 1 hour. Primary and secondary antibodies were used as listed above. Finally, organoids were mounted onto a cell culture dish (Nest, 801001) for imaging using a Leica LSM980 confocal microscope.

### RNA Extraction and qRT‐PCR

Small intestinal organoids were collected and washed once by ice cold PBS. Total RNA was extracted using Trizol reagent (Thermo Fisher Scientific, #15 596 026) and then reverse transcribed into cDNA using HiScript III RT SuperMix for qPCR (+gDNA wiper) (Vazyme, R323‐01). Real‐time PCR was conducted on a CFX96 Real‐Time PCR Detection System (Bio‐rad) using SYBR green fluorescent dye (Vazyme, Q711‐02). Fold changes were calculated by using the delta delta CT method, with *Rpl30* as a reference gene for normalization. Primers were listed in Table S7 (Supporting Information).

### Immunoblotting

The isolated crypt pellets or collected colon cancer cells were lysed in RIPA buffer (Genstar, E122‐01) with protease inhibitors (Roche). And protein extract was quantified using a BCA protein assay kit (Beyotime, P0012S) and 20–40 µg of total protein was separated with 10% SDS PAGE gel under denaturing conditions and was transferred to nitrocellulose membranes (PALL, #66485). The membranes were blocked and incubated with the primary antibodies and subsequent secondary anti‐rabbit or anti‐mouse conjugated antibodies. Anti‐Prmt5, anti‐Cleaved Caspase 3 and anti‐p53 antibodies were used as listed above, and others were as following: rabbit anti‐MMA (CST, #8015), rabbit anti‐SDMA (CST, #13222), rabbit anti‐Rpl10a (Proteintech, #16681‐1‐AP), rabbit anti‐SmB (Proteintech, #16807‐1‐AP), rabbit anti‐Flag (Proteintech, #20543‐1‐AP), rabbit anti‐Fubp1 (Proteintech, #24864‐1‐AP), rabbit anti‐Srf (Proteintech, #16821‐1‐AP) and rabbit anti‐γH2AX (CST, #9718).

### Prmt5‐methylome profiling: Sample Preparation

The small intestinal crypts were isolated from three Prmt5*^fl/fl^* mice (Control) and three *Villin*‐CreERT2; Prmt5*^fl/fl^* mice (KO) after 3 days of TAM treatment. The crypts from the three mice in each group were pooled together for subsequent sample preparation in the Control or KO group. For profiling in small intestinal organoids, EtOH‐ or 4‐OHT‐ treated small intestinal organoids (*Villin*‐CreERT2; Prmt5*^fl/fl^*) were collected and washed once with PBS. Organoids were centrifuged at 1500 rpm for 5 min and supernatant was removed. Then the pellets were incubated with Cell Recovery Solution (Corning, #354253) at 4 °C for 40 min to depolymerize the Matrigel.

Methylated peptides was enriched using a PTMScan Mono‐Methyl Arginine Motif [mme‐RG] Kit (CST, #12235) and PTMScan Symmetric Di‐Methyl Arginine Motif [sdme‐RG] Kit (CST, #13563). Sample preparation, MMA or SDMA peptide enrichment and purification were performed according to manufacturer's instructions of the kits. Briefly, the isolated crypts or collected organoids were lysed in the urea lysis buffer (> 5 mg of total proteins per group). After reduction and alkylation, protein extracts were digested by trypsin. Following the purification of peptides by Sep‐Pak C18 columns (Waters Corporation, WAT020515), a small portion (> 100 µL) of the total peptide solution was kept for later proteome analysis, and the remaining was divided into two equal parts for the subsequent immunoaffinity purification of MMA and SDMA peptides, respectively. After purified by using 20 µL of beads (1/4 of the volume recommended for one sample), the MMA or SDMA peptides were underwent the final concentration and purification by Pierce C18 Tips (Thermo Fisher Scientific, #87784, 100 µL). The proteomic peptides and methylated peptides were then subjected to the following liquid chromatography‐tandem mass spectrometry (LC‐MS/MS) analysis.

### Prmt5‐methylome profiling: LC‐MS/MS Analysis

The proteomic peptides or methylated peptides were analyzed by LC‐MS/MS, combining an Easy‐nLC 1200 connected online to an Orbitrap Fusion Lumos mass spectrometer (Thermo Fisher Scientific), respectively. A 250 mm Acclaim PepMap100 C18 column (Thermo Fisher Scientific) with internal diameter of 75 µm was used to separate the peptides with mobile phase A (0.1% FA in water) and mobile phase B (0.1% FA in 80% ACN) at a 240 min gradient: kept B at 2% for 1min, 2–7% B in 10 min, 7–28% B in 200 min, 28–36% B in 15 min, 36–60% B in 5 min, 60–95% B in 2 min, and then kept B at 95% for 7 min. The flow rate was set as 300 nL min^−1^. For methylated peptides, samples were separated at a 120 min gradient: 2–22% B in 80 min, 22–28% B in 20 min, 28–36% B in 12 min, 36–100% B in 2 min, and then kept B at 100% for 6 min.

The Orbitrap Fusion Lumos mass spectrometer was operated in a data‐dependent acquisition mode. For proteomic peptides, MS1 data were collected by Orbitrap (240,000 resolution, AGC target 100%, maximum injection time 50 ms). Determined charge states between 2 and 7 were required for sequencing and a 90 s dynamic exclusion window was used. The MS2 stage consisted of fragmentation by HCD (normalized collision energy 30%), analyzed using the Ion Trap (rapid scan rate, AGC target 100%, maximum injection time 10 ms, isolation window 1.6 m/z). FAIMS voltage was set as −40, −60, and −80 V and the cycle time was set at 1s per CV. For methylated peptides, MS1 data were collected by Orbitrap (60,000 resolution; AGC target 100%, maximum injection time 120 ms), and the MS2 was analyzed using Orbitrap (15,000 resolution; AGC target 200%, maximum injection time 50 ms, isolation window 1.6 m/z).

### Prmt5‐methylome profiling: Data Processing

Raw data were analyzed by MaxQuant (version 1.6.17.0) search against mouse fasta database downloaded from UniProt (including 17,078 entries, downloaded on 25 March 2021) using the default setting, with label‐free quantification and match between runs functions enabled. Methyl (R) and dimethyl (R) were selected as dynamic modification.

### Immunoprecipitation

Proteins were extracted from isolated small intestinal crypts using ice‐cold lysis buffer containing 20 mm Tris‐Hcl (pH 7.5), 150 mM NaCl, 1 mM EDTA, 1% NP‐40, and protease inhibitors. The extracts were centrifuged at 15,000 × g for 20 min at 4 °C and the supernatants were collected. 20 µL of Protein A/G beads slurry and 1 µg of anti‐SmB antibodies (listed above) or isotype IgG were added into the cell supernatants and incubated overnight at 4 °C. The beads were washed twice with lysis buffer. The immunoprecipitated proteins were eluted by adding 1× SDS loading buffer and heated at 98 °C for 10 min, then analyzed by immunoblotting. The related primary antibodies (listed above) and secondary EasyBlot anti‐Rabbit IgG (HRP) (GenTex, GTX221666‐01)

For immunoprecipitation in colon cancer cell lines, the pLVX3‐Flag‐Sm proteins (SmB, SmD1 or SmD3) or Hnrnpdl plasmids were transfected into the HCT116 cells by PEI, respectively. Lentivirus‐packaged pLVX3‐Flag‐Pnkp plasmids were transduced into SW480 cells. After treatment with 1µM LLY283 for 4 days, the cells were lysed and 5 µL of Anti‐FLAG M2 Magnetic Beads (Merck, M8823) was used per group.

### Bulk RNA Sequencing, Alternative Splicing Analysis and Validation

Total RNA was extracted using Trizol reagent from small intestinal organoids (*Villin‐CreERT2; Prmt5^fl/fl^*) 4 days post‐treatment with EtOH or 4‐OHT, with two biological replicates per group. The mRNA library preparation was performed by using NEBNext Ultra RNA Library Prep Kit for Illumina. Paired‐end sequencing was performed on the Illumina platform with a PE150 strategy by Novogene Bioinformatics Technology Co., Ltd (Beijing, China), based on the required effective library concentration and data amount. mRNA expression analysis was performed using Hisat (version 2.1.0) and Ballgown (version 2.20.0). The differentially expressed genes were identified using EdgeR (version 3.30.3) and DESeq2 (version 1.28.1) software.

For splicing analysis, sequencing reads from the bulk RNA‐sequencing were aligned to UCSC mm10 reference genome, and the alternative splicing events were analyzed by rMATS (version 4.1.2). Differential alternative splicing events with high‐confidence were obtained by filtered with the threshold: | ΔPSI | = | PSI (KO) – PSI (Control) | ≥ 5%, FDR< 0.05, splice junction reads≥ 10. PSI: percentage spliced in, long isoform divided by the sum of both long and short isoforms. The sequences flanking 5′ and 3′ splice site were obtained from the reference genome and splice site strength was detected by MaxEntScan using the maximum entropy scoring model (Table S4, Supporting Information).^[^ ^38^ ^]^

For the validation of alternative splicing events (SE and RI), small intestinal organoids (*Villin‐CreERT2*; *Prmt5^fl/fl^*) were treated with EtOH or 4‐OHT, with three biological replicates per group. Total RNA was extracted 4 days after treatment and then subjected to standard reverse transcription PCR (RT‐PCR) using primer sets specifically targeting the flanking exons of the alternatively spliced exon or retained intron. The PCR products were separated by electrophoresis and the band intensity of long or short isoform was quantified by Image J. Then PSI (band intensity of long isoform divided by the sum of band intensity of both long isoform and short isoform) was calculated for individual genes. The primers used were listed in Table S7 (Supporting Information).

### p53 Knockout in Mouse Intestinal Organoids

The negative control sgRNAs (sgNC) or sgRNAs targeting p53 (sg‐p53 #1 or #2) were inserted into the lentiCRISPRv2 vector. Lentivirus preparation and transduction into small intestinal organoids were carried out as previously described with some modifications.^[^ ^39^ ^]^ Prior to lentivirus infections, organoids were cultured in expansion medium (ENR medium plus 100 ng mL^−1^ Wnt3a, 2 µM CHIR‐99021, 6.67 µM blebbistatin, 5 mM nicotinamide) for 2–3 days. Subsequently, organoids were digested with TrypLE (Gibco, 12604021) in a 37 °C incubator for 5 min, and re‐suspended in 250 µL of expansion medium supplemented with 10 mg mL^−1^ polybrene (Macgene, MC032) and concentrated lentivirus. The mixture was then added onto the pre‐solidified Matrigel in a 48‐well plate and incubated at 37 °C overnight. The following day, the medium was aspirated and 10 µL of Matrigel was added. The organoids were then cultured in expansion medium (minus nicotinamide) for 3 days, followed by ENR medium plus 2 mg mL^−1^ puromycin for selection. The sgRNA sequences used were listed in Table S7 (Supporting Information).

### Bioinformatics Analysis

Prmt5 methylation motif analysis was conducted using the IceLogo online tool,^[^ ^40^ ^]^ by taking 10 amino acids upstream and downstream of the methylated R site. GO, KEGG pathway, and protein‐protein interaction network were analyzed using the Metascape web server.^[^ ^41^ ^]^ Venn diagrams were conducted using a web server EVenn.^[^ ^42^ ^]^ Integrative genomics viewer (IGV) was used to visualize the RNA‐sequencing data.^[^ ^43^ ^]^ Protein conserved domains were searched in CDD database (v3.20) web server.^[^ ^44^ ^]^ GSEA on p53 signaling pathway was performed using an online tool (https://www.bioinformatics.com.cn).

### Statistics

All experiments presented in this study were independently repeated at least two times, unless explicitly indicated in the main text or figure legends. Exact numbers of mice, fields, and organoids are shown in the figure legends. Crypt, organoid, or cell number difference between two groups were compared using Mann‐Whitney (two‐tailed) U‐test. Difference between two groups in mouse body mass at individual days and in all PCR assays were compared using unpaired Student's t‐test. All data shown in graphs represent mean ± SD, *p< 0.05, **p< 0.01, ***p< 0.001. All statistical analysis were performed with Graph‐Pad Prism (version 8.0).

## Conflict of Interest

The authors declare no conflict of interest.

## Author Contributions

L.L. and Y.C. conceived the experiments, analyzed the data, and wrote the manuscript. L.L. conducted all the experiments. H.Z. gave advice on experiments. Z.Z. performed alternative splicing analysis by rMATS. X.W. performed the bulk RNA‐sequencing analysis. L.L., Y.X., and S.H. provided assistance with mice experiments and organoid experiments.

## Supporting information



Supporting Information

Supplemental Table 1

Supplemental Table 2

Supplemental Table 3

Supplemental Table 4

Supplemental Table 5

Supplemental Table 6

Supplemental Table 7

## Data Availability

All the single‐cell level analyses were conducted using publicly available single‐cell RNA‐sequencing data from wild type mouse small intestinal epithelium (accession GSE186913). The raw bulk RNA‐sequencing data in this study was deposited in the Genome Sequence Archive in National Genomics Data Center, China National Center for Bioinformation/ Beijing Institute of Genomics, Chinese Academy of Sciences (GSA accession number: CRA013488) that are publicly accessible at https://ngdc.cncb.ac.cn/gsa.^[^ ^45^ ^]^
