# Supplementary material for: PRMT5 Maintains Homeostasis of the Intestinal Epithelium by Modulating Cell Proliferation and Survival
Source: Adv Sci (Weinh). 2025 Feb 3;12(12):2415559. doi: 10.1002/advs.202415559 (PMC11948081; doi:10.1002/advs.202415559)
Supplement: Supplementary file 1 — Supporting Information [file ADVS-12-2415559-s005.docx]

Supporting Information

**PRMT5 maintains homeostasis of the intestinal epithelium by modulating cell proliferation and survival**

Leilei Li, Zhe Zhang, Xu Wang, Haiyong Zhao, Liansheng Liu, Yanhui Xiao, Shan Hua, and Ye-Guang Chen

**This word file includes:**

Figures S1 to S7

**Other Supplementary Materials for this manuscript include the following:**

Table S1 to S7


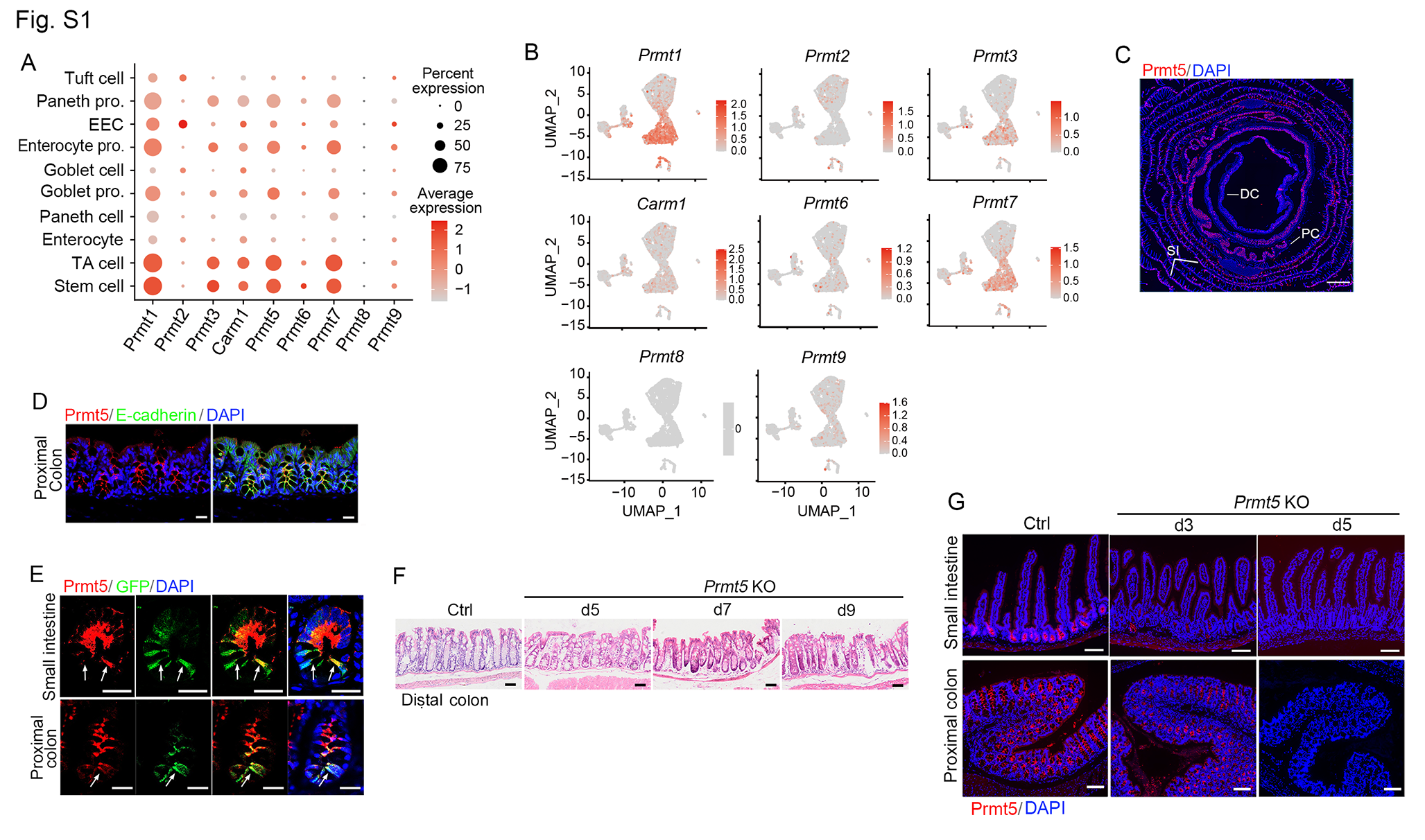


**Figure S1. Prmt5 is required for intestinal homeostatic maintenance.**

(A) Dot plot of Prmt family expression in different small intestinal cell types. Dot color saturation indicates the average expression level (scaled by Z-score), and the dot size indicates the percentage of cells expressing the gene.

(B) UMAP visualization of Prmt family expression.

(C) Immunofluorescence staining of Prmt5 in the intestinal “Swiss rolls” from small intestine to distal colon. SI, small intestine; PC, proximal colon; DC distal colon.

(D) Prmt5 and E-cadherin staining in proximal colon.

(E) Immunofluorescence staining of Prmt5 and GFP in small intestinal and proximal colon crypts from *Villin-CreERT2*; *Lgr5-EGFP-IRES-CreERT2*; *Prmt5^fl/fl^* mice. Arrows indicated the cells co-stained with Prmt5 and GFP.

(F) H&E staining in distal colon at the indicated time points.

(G) Immunofluorescence staining of Prmt5 in small intestine and proximal colon at 3 or 5 dpt.

Scale bars: 1 mm (C), 20 μm (D, E), 50 μm (F), 100 μm (G). Nuclei were counter-stained with DAPI.


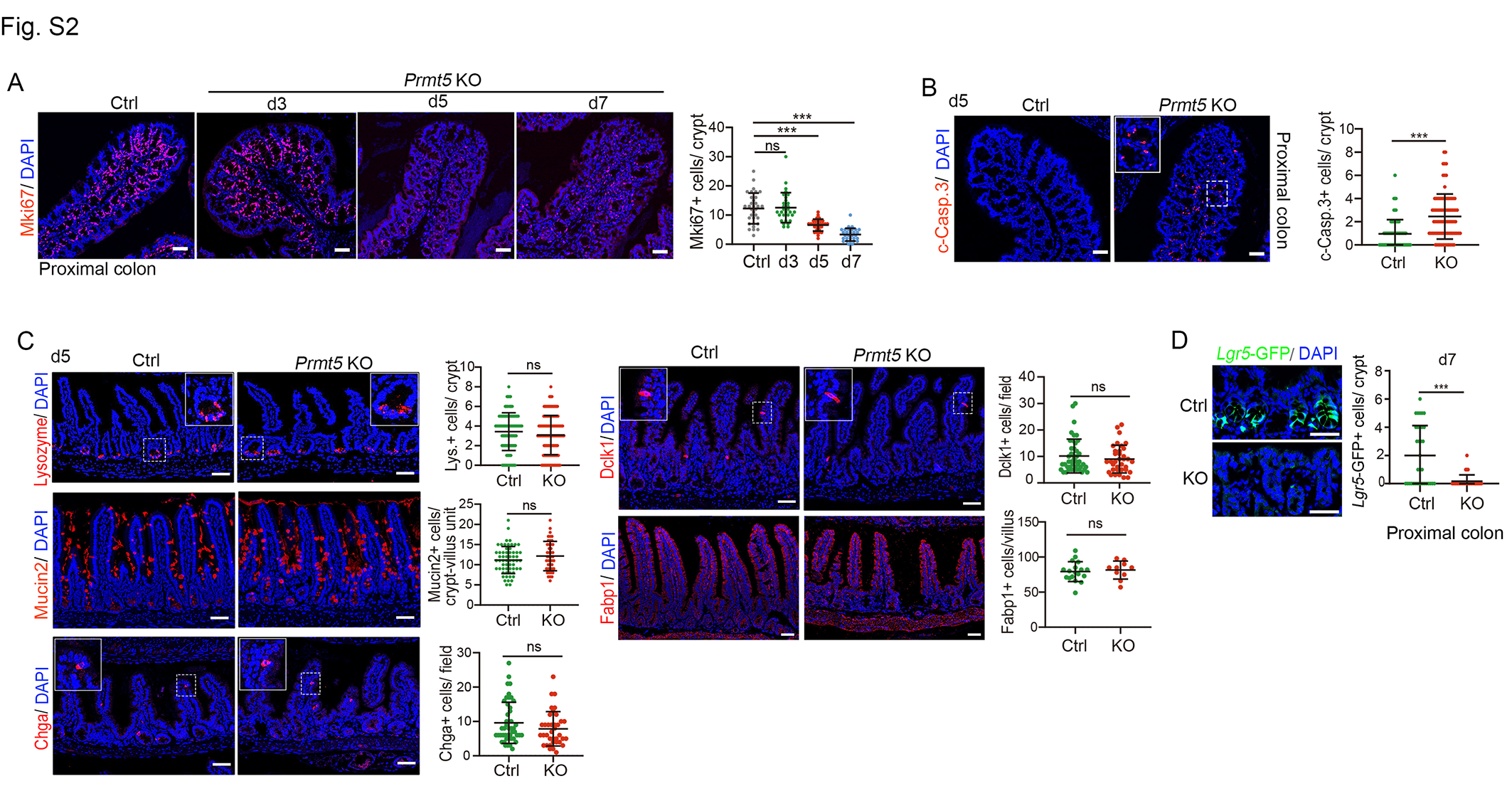


**Figure S2. Prmt5 is essential for cell proliferation and intestinal stem cell maintenance.**

(A) Immunofluorescence images and quantification of Mki67^+^ cells in proximal colon at indicated times. n = 30-33 random crypts from 2-3 mice/ group.

(B) Immunofluorescence images and quantification of c-Casp.3^+^ cells in proximal colon at 5 dpt. n = 71-89 random crypts from 3 mice/ group.

(C) Immunofluorescence images or quantification of Lysozyme^+^ cells (n = 152-169 crypts from 3 mice/group), Mucin2^+^ cells (n = 45-60 crypt-villus units from 3 mice/group), Chga^+^ cells (n = 37-47 fields from 3 mice/group), Dclk1^+^ cells (n = 35-44 fields from 3 mice/group), or Fabp1^+^ cells (n = 10-20 villus from 2-3 mice/group) in small intestine at 5 dpt.

(D) Immunofluorescence images and quantification of *Lgr5*-GFP^+^ cells in proximal colon from Control (*Villin-CreERT2*; *Lgr5-EGFP-IRES-CreERT2*) or Prmt5 KO (*Villin-CreERT2*; *Lgr5-EGFP-IRES-CreERT2*; *Prmt5^fl/fl^*) mice at 7 dpt. n = 30-34 random crypts from 3 mice/ group.

All the data represent mean ± SD, ***p < 0.001, **p < 0.01, *p < 0.05, ns= not significant, Mann-Whitney (two-tailed) U-test. Scale bars: 50 μm (A-D). Nuclei were counter-stained with DAPI.


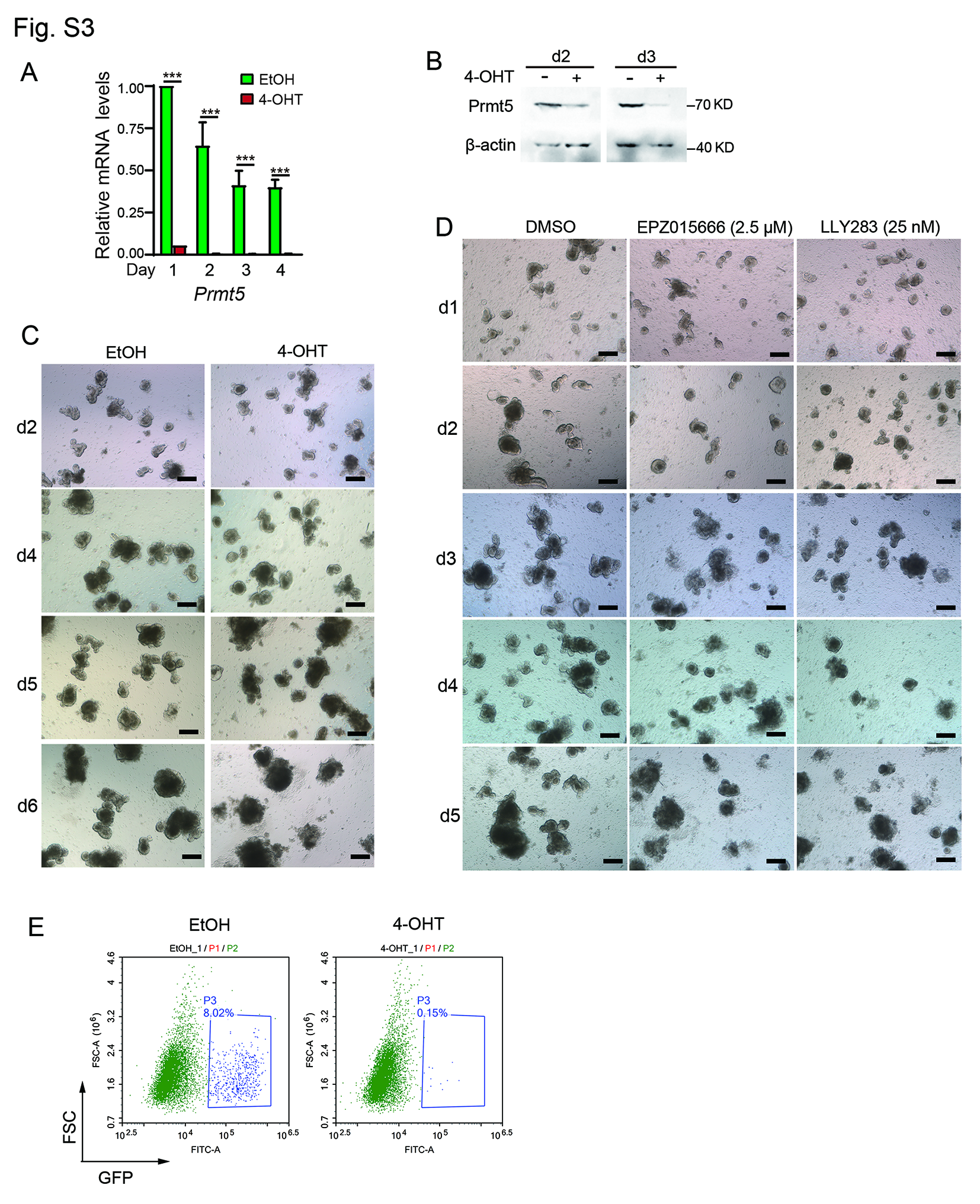


**Figure S3. Prmt5 is necessary for cell proliferation and survival in intestinal organoids.**

(A) Prmt5 mRNA expression was examined by RT-qPCR in organoids (*Villin-CreERT2*; *Prmt5^fl/fl^*) at indicated time points post EtOH or 4-OHT treatment. Data represent mean ± SD, ***p < 0.001, from one of three independent experiments. Unpaired student-t test.

(B) Prmt5 protein levels were examined by immunoblotting in organoids (*Villin-CreERT2*; *Prmt5^fl/fl^*) at indicated time points post EtOH or 4-OHT treatment

(C) Images of *Villin-CreERT2*; *Prmt5^fl/fl^* organoids at indicated time points following EtOH or 4-OHT treatment. Scale bars: 200 μm.

(D) Images of Villin-CreERT2; Prmt5^fl/fl^ organoids after continuous treatment with compounds for indicated days. Scale bars: 200 μm.

(E) Small intestinal organoids (*Lgr5-EGFP-IRES-CreERT2*; *Prmt5^fl/fl^*) were treated with EtOH or 1 μΜ 4-OHT for 2 days and the *Lgr5*-GFP^+^ cells were quantified by flow cytometry at day 10 post treatment.


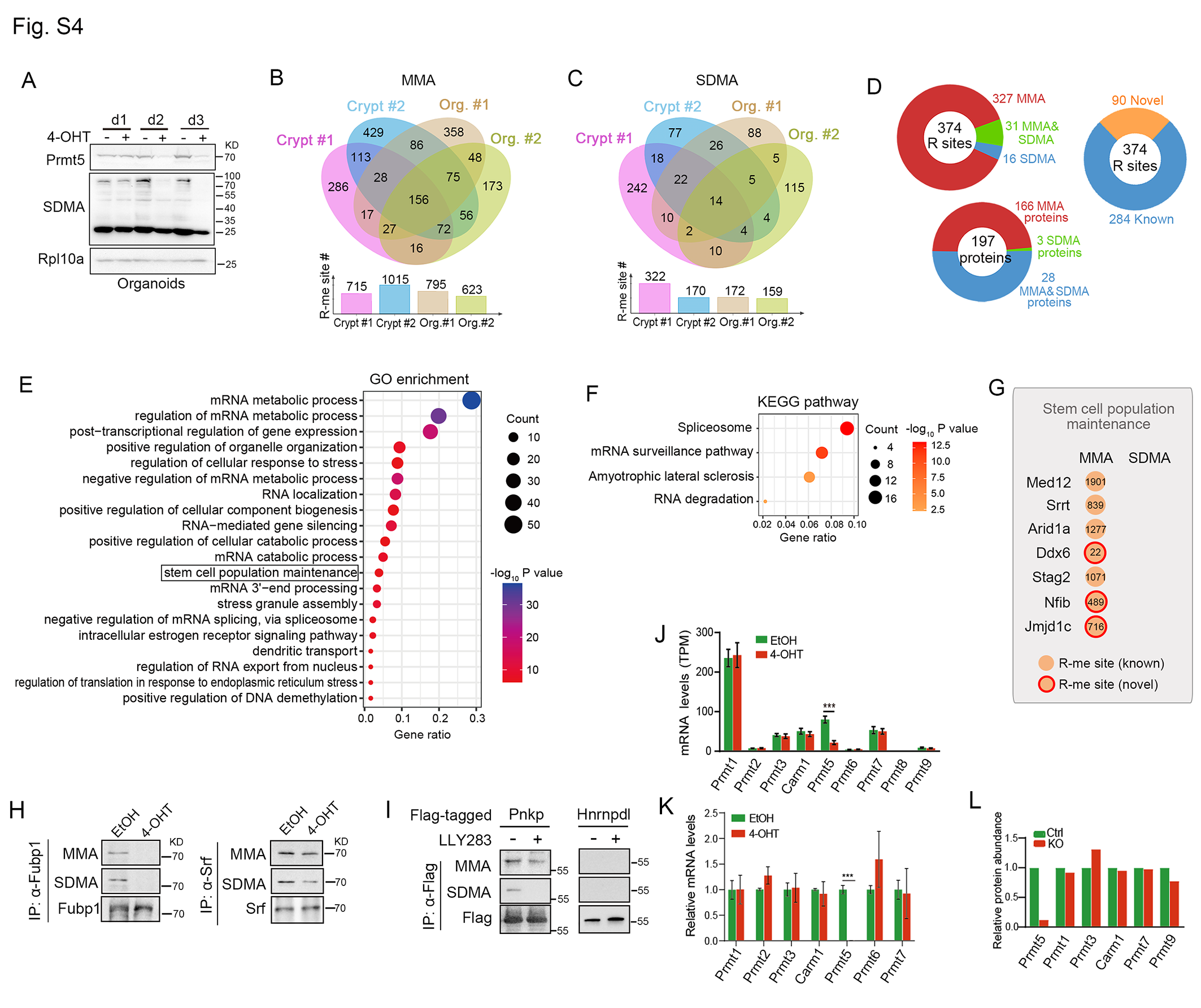


**Figure S4. Arginine-methylated proteins in mouse intestinal epithelium.**

(A) The global SDMA was immunoblotted in small intestinal organoids (*Villin-CreERT2*; *Prmt5^fl/fl^*) at 1-3 days post EtOH or 4-OHT treatment. Rpl10a, reference protein.

(B, C) Venn diagram shows the number of identified MMA (B) and SDMA (C) sites in the Control group of four independent profiling assays. Upper, overlap among four independent assays; Lower, R-me number of four assays. The diagram were plotted by a web server EVenn.

(D) Pie charts show the overlapped modified R sites and corresponding proteins. Upper, the number of R sites with different modification types (left) and the number of known or novel methylated R sites (right); Lower, the number of corresponding proteins with different modification types.

(E, F) Gene ontology (GO) (E) and KEGG pathway (F) enrichments of methylated proteins in Control group were analyzed using Metascape.

(G) Arginine-methylated proteins involved in stem cell population maintenance are listed. Only the R-me sites present in at least three methylome profilings are listed. The specific residues are shown inside the circles. Outlined circles are novel sites. Known arginine methylated sites (mouse) were retrieved from http://www.phosphosite.org.

(H) Fubp1 and Srf proteins were immunoprecipitated and the MMA and SDMA modifications were detected by immunoblotting in *Villin-CreERT2*; *Prmt5^fl/fl^* intestinal organoids upon EtOH or 4-OHT treatment.

(I) Flag-tagged Pnkp or Hnrnpdl was ectopically expressed and immunoprecipitated in HCT116 or SW480 colon cancer cells treated with vehicle or 1μM LLY283, then MMA and SDMA were detected by immunoblotting.

(J) The mRNA levels of Prmt family from bulk RNA-sequencing in *Villin-CreERT2; Prmt5^fl/fl^* organoids post EtOH or 4-OHT treatment. TPM, transcript per kilobase per million mapped reads.

(K) The relative mRNA levels of Prmt family were detected by RT-qPCR in *Villin-CreERT2; Prmt5^fl/fl^* organoids post EtOH or 4-OHT treatment.

(L) Relative protein abundance of Prmts in control and Prmt5 KO group. The abundance of individual protein represents the average protein abundance from four methylome profilings.

**
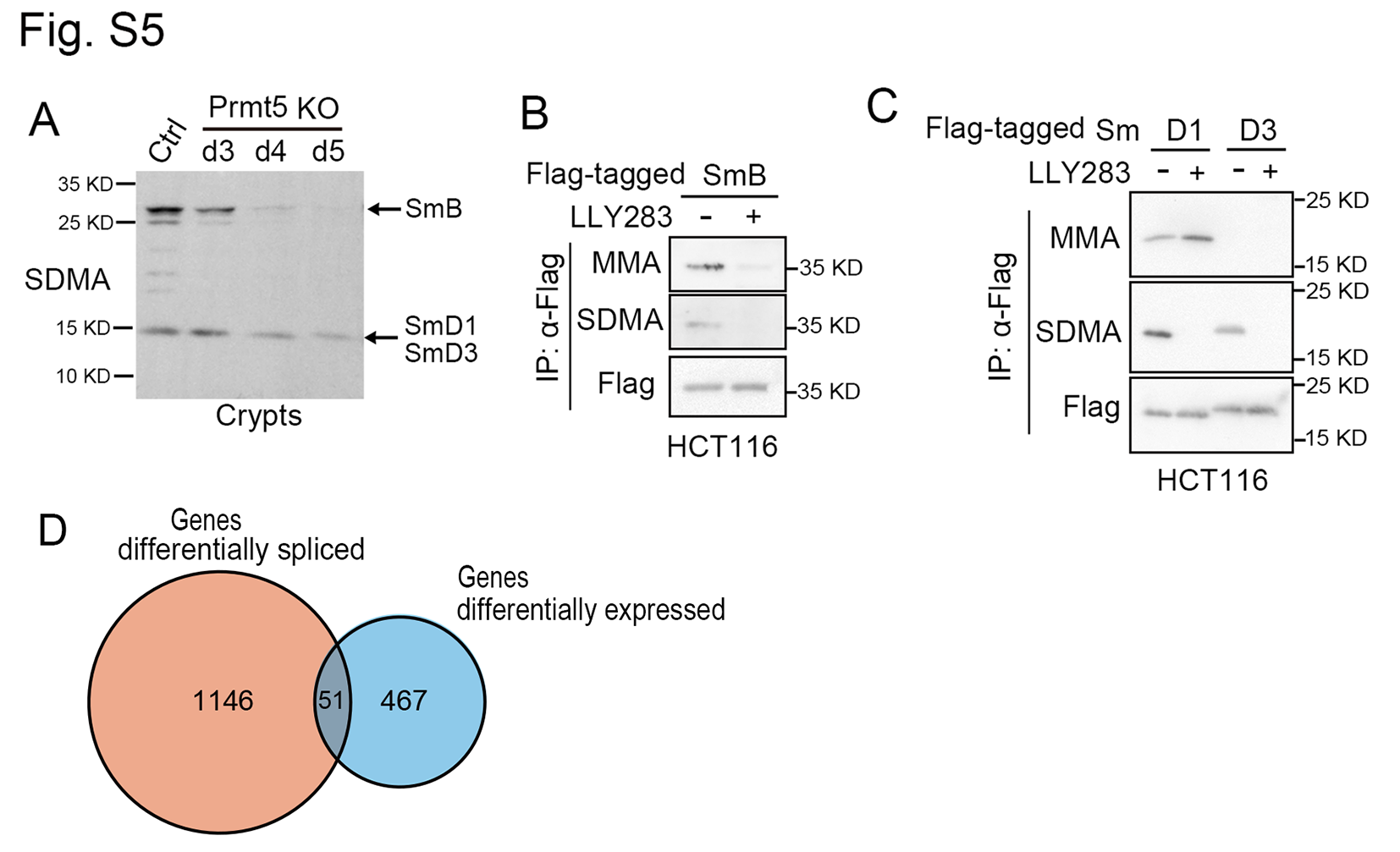
**

**Figure S5. Prmt5 deletion decreases arginine methylation of Sm proteins.**

(A) Immunoblot showed the gradually decreased SDMA modification of SmB (~ 25KD), SmD1 (~ 13KD), and SmD3 (~ 14KD) at indicated times upon *Prmt5* KO in mouse small intestinal crypts.

(B, C) Flag-tagged SmB, SmD1 or SmD3 was ectopically expressed and immunoprecipitated, then SDMA was detected by immunoblotting in HCT116 cells treated with vehicle or 1μM LLY283.

(D) Venn diagram shows a minimal portion of overlapped genes between 1197 differentially spliced genes and 518 differentially expressed genes (Fold change ≥1.5, p≤0.05) in bulk RNA-sequencing data.


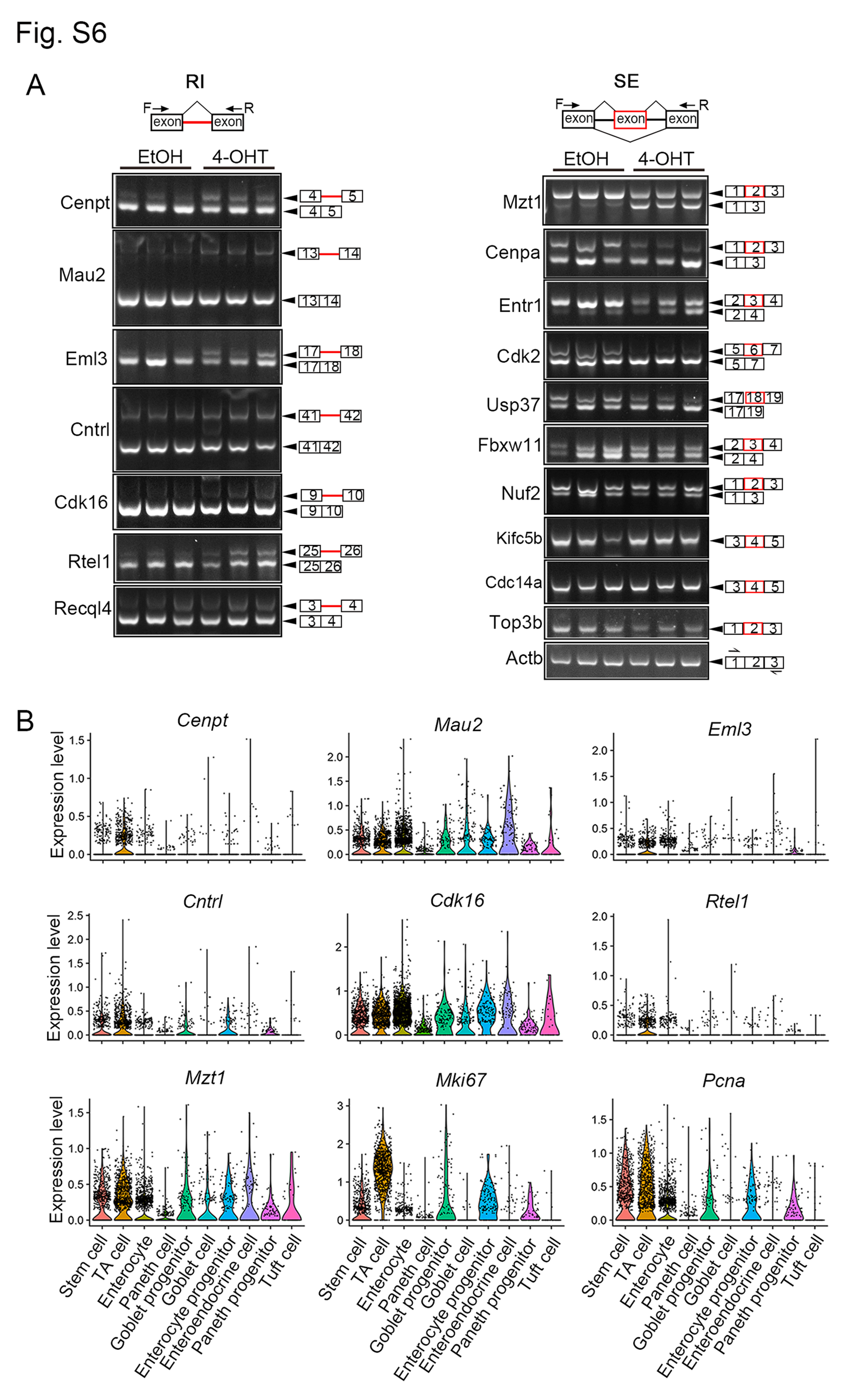


**Figure S6. Prmt5 loss disrupts splicing of a subset of mitotic cell cycle-related genes.**

(A) Disrupted RI and SE events of cell cycle-related genes were validated by RT-PCR in *Villin-CreERT2*; *Prmt5 ^fl/fl^* organoids treated with EtOH or 4-OHT. Three biological replicates/ group. The band show the long isoform (intron or exon included) or the short isoform (intron or exon excluded) for every gene detected. Red line or red box represents the retained intron or skipping exon.

(B) Volcano plot shows the expression of cell cycle genes in small intestinal epithelium in scRNA-sequencing analysis.

**
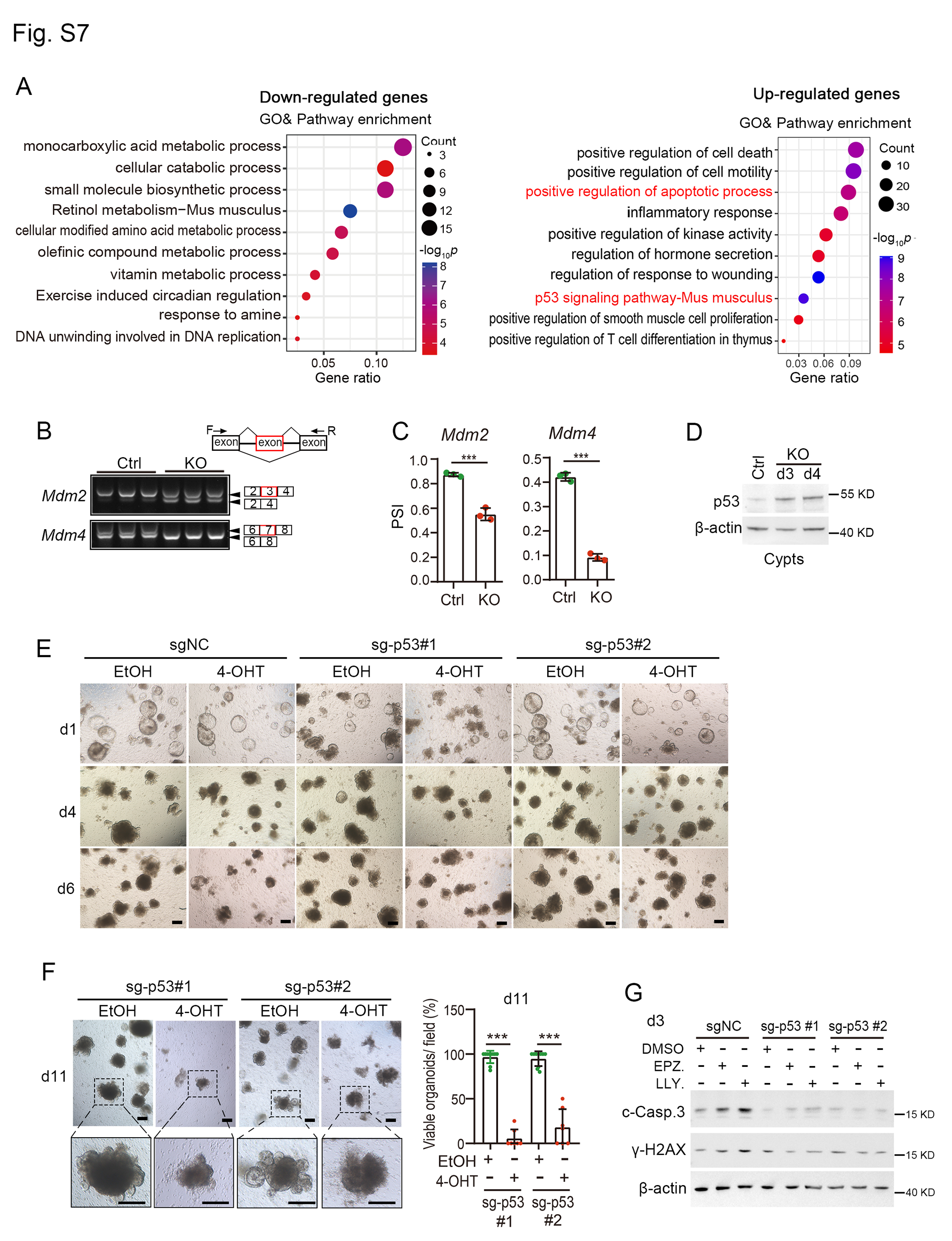
**

**Figure S7 Prmt5 deletion activates p53 apoptotic signaling pathway.**

(A) GO& Pathway enrichment analysis was performed on the downregulated (147 genes) or upregulated genes (371 genes) (fold change ≥1.5, p≤0.05) identified by bulk RNA-sequencing 4 days following 4-OHT treatment in *Villin-CreERT2*; *Prmt5 ^fl/fl^* organoids. Metascape was used for the analysis.

(B, C) Validation (B) and quantification (C) of the SE event in the *Mdm2* or *Mdm4* transcript.

(D) Indicated proteins were detected by immunoblotting in isolated intestinal crypts from *Prmt5 ^fl/f^* (control) or *Villin-CreERT2*; *Prmt5 ^fl/fl^* (KO) mice treated with tamoxifen. Data were from one of three independent experiments.

(E) Images of organoids at indicated time in control (sgNC) or p53 KO (sg-p53) organoids (*Villin-CreERT2*; *Prmt5 ^fl/fl^*) following EtOH or 4-OHT treatment. n ≥ 10 fields/ group. Scal bar, 200 μm. Data were from one of three independent experiments.

(F) Images and quantification of viable organoids (relative to total organoids/ field) in control or p53 KO organoids (*Villin-CreERT2*; *Prmt5 ^fl/fl^*) 11 days post EtOH or 4-OHT treatment. n ≥ 10 fields/ group. Scal bar, 200 μm. ***p < 0.001, unpaired student t-test. Data were from one of three independent experiments.

(G) Indicated proteins were detected by immunoblotting in the control or p53 KO organoids treated with vehicle (DMSO), EPZ015666 or LLY283 for 3 days. Data represent one of three independent experiments.

All the data represent mean ± SD. ***p < 0.001, **p < 0.01, *p < 0.05, Mann-Whitney (two-tailed) U-test (F), unpaired student-t test (C). Scale bars: 200 μm (E, F).

**Table S1.** The proteomes and methylomes in four Prmt5-methylome profiling assays.

**Table S2.** The R-me sites and related proteins present in at least three of the Prmt5-methylome profiling assays.

**Table S3.** The differential alternative splicing events upon Prmt5 KO in organoids.

**Table S4.** The 5'SS, 3'SS and their strength in RIs and SEs.

**Table S5.** The roles of seven cell cycle-related genes

**Table S6.** The bulk RNA sequencing in intestinal organoids upon Prmt5 KO.

**Table S7.** The primers and sgRNAs used in this study.
